# P09-09 Hybrid work and temporal patterns of sedentary behavior in a 24-hour compositional perspective

**DOI:** 10.1093/eurpub/ckac095.139

**Published:** 2022-08-29

**Authors:** David Hallman, Leticia Januario, Svend Erik Mathiassen, Marina Heiden, Viktoria Wahlström

**Affiliations:** 1 Occupational health sciences and psychology, University of Gävle, Gavle, Sweden; 2 University of Gävle, Gävle, Sweden; 3 Umeå University, Umeå, Sweden

**Keywords:** Working from home, sitting, time-in bed, compositional data analysis

## Abstract

**Background:**

During the COVID-19 pandemic, many white-collar workers were requested to exclusively work from home (WFH), which may have affected their sedentary behavior. In Sweden, having less severe restrictions than many other countries, workers were allowed to alternate between WFH and work at the office (WAO), so called hybrid work. Understanding how hybrid work influences total sedentary behavior and its temporal distribution is an important issue for future health promotion. This study aimed to investigate to what extent office workers changed their temporal pattern of sedentary behavior during days WFH compared to WAO, considering age and gender as potential moderators.

**Methods:**

Data were collected from May to December 2020 in office workers (n = 199). Their mean age was 42 (*SD* 10) years and 55% were women. Physical behaviors were measured using a thigh-worn accelerometer (AxivityAX3) for seven consecutive days. A diary identified working hours, time-in-bed and days WFH or WAO. Time-use was classified as short (0-5 min), moderate (5-30 min) and long bouts (>30 min) of sedentary behavior (SB), non-SB, and time-in-bed during workdays (WAO and WFH) and non-workdays. We used Compositional data analysis to express data as 24-hour compositions and linear mixed models to estimate difference in 24-hour compositions between day types (within worker), including age and gender as covariates and moderators.

**Results:**

We found that workdays (WFH and WAO) were associated with proportionally less time-in-bed relative to time awake, more time SB relative to non-SB, and more time in longer relative to shorter sedentary bouts, compared to non-workdays (all p > 0.001). WFH was associated with more time-in-bed relative to awake and more SB relative to non-SB than WAO (p > 0.05), but the differences for sedentary bouts were not significant. Younger workers and women had more SB, and women accumulated more time than men in longer relative to shorter bouts of SB. However, age and gender did not affect differences between day types.

**Conclusions:**

Working from home influenced 24-hour time-use in office workers by increasing sedentary behavior in total, while its temporal pattern was unchanged. Results contribute to evidence that can support organizational policies on hybrid work.

